# Epidermal growth factor regulation by autophagy‐mediated lncRNA H19 in murine intestinal tract after severe burn

**DOI:** 10.1111/jcmm.15262

**Published:** 2020-04-16

**Authors:** Cuijie Li, Mengmeng Zhuang, Bo Zhu, Ye Li, Wenwen Zhang, Hao Yan, Pan Zhang, Dan Li, Juan Yang, Yuan Sun, Haijun Chen, Qingwei Cui, Peisheng Jin, Yong Sun

**Affiliations:** ^1^ Department of Burn Surgery The Affiliated Huaihai Hospital of Xuzhou Medical University Xuzhou China; ^2^ Department of Burn Surgery The 71st Group Army Hospital of PLA Xuzhou China; ^3^ Department of Plastic Surgery the Affiliated Hospital of Xuzhou Medical University Xuzhou China

**Keywords:** autophagy, burn, intestine, let‐7g, lncRNA H19

## Abstract

To investigate the regulation of epidermal growth factor (EGF) by autophagy‐mediated long non‐coding RNA (lncRNA) H19 in the intestinal tracts of severely burned mice. C57BL/6J mice received third‐degree burns to 30% of the total body surface area. Rapamycin and 3‐methyladenine (3‐MA) were used to activate and inhibit autophagy, and the changes in LC3 and Beclin1 levels were assessed by Western blotting. The effect of autophagy on lncRNA H19 was detected by qRT‐PCR. Adenovirus‐mediated overexpression of lncRNA H19 in IEC‐6 cells was used to assess the effects of lncRNA H19 on EGF and let‐7g via bioinformatics analysis, Western blotting and qRT‐PCR. let‐7g mimic/inhibitor was used to overexpress/inhibit let‐7g, and qRT‐PCR and Western blotting were used to detect the effects of let‐7g on EGF. The expression levels of LC3‐II, Beclin1 and lncRNA H19 were increased in intestinal tissues and IEC‐6 cells after rapamycin treatment but were reversed after 3‐MA treatment. LC3‐II, Beclin1 and lncRNA H19 levels increased in intestinal tissues after the burn, and these increases were more significant after rapamycin treatment but decreased after 3‐MA treatment. The lncRNA H19 overexpression in IEC‐6 cells resulted in increased and decreased expression levels of EGF and let‐7g, respectively. Furthermore, overexpression and inhibition of let‐7g resulted in decreased and increased expression of EGF, respectively. Taken together, intestinal autophagy is activated after a serious burn, which can increase the transcription level of lncRNA H19. lncRNA H19 may regulate the repair of EGF via let‐7g following intestinal mucosa injury after a burn.

## 1 INTRODUCTION

The small intestine has the largest surface area among all organs in the body and is the largest bacterial storage organ in the human body. The intestinal mucosa requires symbiotic bacteria to maintain its dynamic balance under latent adverse signals and adverse external conditions, such as the presence of undesirable microorganisms, toxins and food antigens, by regulating the inflammatory response. One of the main functions of the intestinal mucosa is to form a barrier between the stroma and intestinal contents, and a single layer of intestinal epithelial cells serves as the dynamic interface between the host and external environment.^1^ At the same time, the intestinal tract is considered one of the central organs of traumatic stress. During a burn, trauma and major surgery, the stress, tissue ischaemia and hypoxia as well as infection and other factors can result in damage to the intestinal mucosal barrier.^2^ Such damage is the pathophysiological basis for post‐traumatic enterogenous infection, enterogenous hypermetabolism, systemic inflammatory response syndrome and subsequent multiple organ dysfunction.^3^ Therefore, the intestinal mucosa plays an important role in the maintenance of human health. Epidermal growth factor (EGF) is a protein with 53 amino acids and is a ligand for the epidermal growth factor receptor.^4^ Ligand binding activates a plethora of downstream signalling cascades involved in cellular proliferation,^5^, ^6^ migration^6^ and survival.^7^, ^8^, ^9^ A large number of studies have shown that EGF plays an important role in promoting restoration of the intestinal mucosa.

Autophagy is a continuous ‘degradation–renewal’ cycle in which damaged and/or redundant organelles, cell components and unfolded proteins are encapsulated by a bilayer membrane and then decomposed into basic biomacromolecules.^10^ This provides the nutritional and metabolic requirements of the cells. Under normal circumstances, autophagy plays a role in the survival, metabolism, growth, development and differentiation of cells.^10^, ^11^ When the body is stimulated by a series of factors, such as stress, ischaemia and hypoxia damage, hunger, lack of nutrition, tumour and neurodegenerative disease,^12^, ^13^ autophagy promotes cell survival by removing damaged proteins and organelles in cells, and also participates in the elimination of exogenous substances and pathogens.^14^, ^15^ Autophagy consists of four key steps: initiation, vesicle nucleation, vesicle elongation, and finally fusion and degradation.^16^, ^17^ Beclin1 (a homolog of yeast Atg6 in mammals) participates in the process of vesicle nucleation.^17^, ^18^, ^19^ The microtubule‐associated protein 1 light chain 3 (LC3; a homolog of yeast Atg8 in mammals) is cleaved by ATG4 protease to form LC3‐I, which is then conjugated to phosphatidylethanolamine to form LC3‐II and subsequently participates in the formation and elongation of autophagosomes.^17^, ^20^, ^21^ Therefore, many researchers use Beclin1 and LC3‐II as markers to monitor autophagy.

In recent years, long non‐coding RNA (lncRNA) has attracted increasing attention. lncRNAs are RNAs that are longer than 200 nucleotides and lack a specific complete open reading frame and thus have no protein‐coding function^22^ and is generally less conserved.^23^ lncRNAs are widely expressed in various tissues and have diverse functions. At present, it is believed that the functional mechanisms mainly include interfering with the expression of downstream genes, influencing the gene transfer of coding proteins and regulating protein functions. Gene expression can be regulated at multiple levels,^24^, ^25^, ^26^ with important links to many diseases. Studies have shown that the lncRNA SPRY4‐IT1 is involved in regulating the repair process of the gastrointestinal mucosal barrier.^27^ The lncRNA H19 gene belongs to a highly conserved imprinted gene cluster that plays important roles in embryonal development and growth control.^28^ Furthermore, a role for H19 acting either as a tumour suppressor or an oncogene has been suggested.^29^, ^30^ However, whether H19 can promote intestinal barrier repair remains poorly understood. MicroRNAs (miRNAs) are a class of endogenous, non‐coding RNA molecules of approximately 18‐25 nucleotides in length.^31^ MiRNAs can regulate protein expression by inhibiting or inducing the degradation of messenger RNAs (mRNAs) by specifically binding to the 3’ untranslated region (UTR) of the mRNAs.^28^, ^31^, ^32^ The Let‐7 family is one of the most widely studied miRNAs, and H19 has been shown to act as an endogenous let‐7g sponge to increase the expression of target genes.^28^, ^33^

Burns can be classified as a systemic disease. When ischaemia and hypoxia occur in the early stages of burns, the structure and barrier functions of the intestine are impaired, and the permeability of the intestine is increased, resulting in intestinal infection. Therefore, the intestinal mucosa plays an important role in the maintenance of human health. The aim of this study was to evaluate the effects of autophagy‐mediated lncRNA H19 in the mouse intestinal tract after severe burns. We report the expression of autophagy‐related proteins after burns and the lncRNA H19 content after autophagy intervention in vitro and in vivo. Then, we explored the effects of lncRNA H19 on let‐7g and EGF by inducing the overexpression of lncRNA H19. Moreover, the expression levels of let‐7g and EGF in severely burned mice were assayed by qRT‐PCR and Western blotting.

## 2 MATERIALS AND METHODS

### 2.1 Animal handling and third‐degree burn model

Clean healthy adult C57BL/6J mice (n = 36) weighing approximately 22 g were purchased from the Jinan Pengyue Experimental Animal Center, Shandong, China. The mice were adapted by feeding using the experimental ventilation and temperature conditions for one week. This experimental programme was approved by the Jiangsu Animal Experiment Ethics Committee and complied with the Guide for Laboratory Animal Care and Use.

To establish the third‐degree burn model,^21^ the mice were fasted for 12 hours with unrestricted access to drinking water. The mice were anaesthetized with an intraperitoneal injection of 1% sodium pentobarbital (40 mg/kg), the back hair was removed with scissors, and the back was placed in hot water at 100°C for 10 seconds, which produced a third‐degree burn covering 30% of the total body surface area. Sodium lactate Ringer's injection (50 mL/kg) was administered immediately via intraperitoneal injection as an anti‐shock treatment. The back of each mouse was kept free of infection, and the mice were kept warm and had free access to water. Sham mice were anaesthetized, depilated as described above and exposed to warm water at 37°C for 10 seconds.

The mice were randomly divided into six groups (n = 6 per group): control group (C), rapamycin group (5 mg/kg rapamycin via intraperitoneal injection), 3‐methyladenine group (3‐MA, 15 mg/kg via intraperitoneal injection), burn group (B), B + Rapamycin group (intraperitoneal injection of 5 mg/kg rapamycin 30 min before the burn) and B + 3‐MA group (intraperitoneal injection of 15 mg/kg 3‐MA 30 minutes before the burn). Rapamycin (B20714; Shanghai Yuanye Bio‐Technology) is an activator of autophagy, whereas 3‐MA (B25357; Shanghai Yuanye Bio‐Technology) is an inhibitor of autophagy.^19^

### 2.2 Cell culture and treatment

IEC‐6 rat small intestinal epithelial cells were purchased from Shanghai Zhongqiao Xinzhou Biotechnology (China). The cells were cultured in Dulbecco's modified Eagle's medium (ExCell) containing 10% foetal bovine serum (FBS; GE, USA), 100 U/mL penicillin and 100 mg/mL streptomycin (both from Beyotime) and cultured in an incubator at 37°C in an atmosphere of 5% CO_2_. Cells were passaged every 2 to 3 days. Logarithmic growth phase cells were used for the experiments.

The IEC‐6 cells were divided into three groups: control group (C, untreated), Rapamycin group (0.5 mM/L) and 3‐MA group (5 mM/L).^34^

### 2.3 Transfection of H19 adenovirus

IEC‐6 cells were inoculated in a 10‐cm cell culture dish at a density of 1 × 10^5^ cells/mL and cultured at 37°C in a 5% CO_2_ incubator. Adenovirus infection was carried out when the cells were approximately 70% confluent. The small‐volume method of infection was used. First, half of the complete medium (4‐5 mL) was added and an appropriate amount of target virus and negative control virus (multiplicity of infection of 100) were added in different petri dishes according to the virus titre. After 4 to 8 hours, the volume of the complete medium was supplemented to 8 to 10 mL. The expression of green fluorescent protein could be observed 24 hours after the infection and reached a peak at 48 hours.

### 2.4 let‐7g gene overexpression and silencing

The cells were digested with trypsin 18 to 24 hours before transfection and resuspended in medium containing FBS but without penicillin and streptomycin. After adjusting the cell concentration, the cells were inoculated in wells of 6‐well plates (2 × 10^5^ cells per well). After overnight culture, the cells were 30%‐50% confluent. Opti‐MEM medium without FBS and penicillin and streptomycin were used to dilute the X‐tremeGENE siRNA Transfection Reagent (Sigma). Similarly, the Opti‐MEM medium (Thermo Fisher Scientific) was used to dilute the gene to be transfected. Within 5 minutes, the diluted transfection reagent was mixed with the gene. The ratio of transfection reagent (mL) to gene (mg) was 5:1. The transfection complex was incubated for 15 to 20 minutes and then added to the IEC‐6 cells cultured in wells of a 6‐well plate for transfection. The let‐7g mimic/inhibitor was transfected into IEC‐6 (20 nmol/L) for overexpression or silencing for 48 to 72 hours for subsequent experiments.

### 2.5 Western blotting

RIPA lysate and phenylmethylsulphonyl fluoride were used to extract proteins from intestinal tissue samples and IEC‐6 cells. The protein concentration was determined by a BCA protein quantitative kit (Beyotime, P0012S). The proteins were separated by SDS‐PAGE and transferred to a polyvinylidene fluoride membrane. After blocking with 5% skim milk for 2 hours, primary antibody to glyceraldehyde‐3‐phosphate dehydrogenase (GAPDH, 1:5000, 10494‐1‐AP; Proteintech), LC3 (1:1000, 2775S; Cell Signaling Technology), Beclin1 (1:1000, 3738S; Cell Signaling Technology) and EGF (1:200, sc‐374255; Santa Cruz Biotechnology) was added and incubated overnight at 4°C. After washing three times with Tris‐buffered saline containing Tween‐20, secondary antibody (ZB‐2301; ZSGB‐BIO) was added and incubated for 2 hours at room temperature. Protein bands were detected using an enhanced chemiluminescent ECL kit (Beyotime, P0018FS).

### 2.6 qRT‐PCR

Total RNA was extracted from intestinal samples using TRIzol reagent (TIANGEN, DP405, China). First‐strand cDNA was synthesized using 2 µg total RNA and a TIANScript RT Kit (TIANGEN, KR104). The microRNA (miRNA) first‐strand cDNA was synthesized using 2 µg total RNA and miRNA First‐strand Synthesis Kit (TIANGEN, KR211). The primers for Actin were 5′‐GGGCTATGCTCTCCCTCACG‐3′ (forward) and 5′‐ TGATGTCACGCACGATTTCC −3′ (reverse). The primers for H19 were 5′‐ GTCGATTGCACTGGTTTGGA‐3′ (forward) and 5′‐ CACACCCAGTTGCCCTCAGA −3′ (reverse). The primers for EGF were 5′‐GTGCTCGTATGTGCTCTTGTG‐3′ (forward) and 5′‐ TCCTTCCCAGTGTGTTTGTTTG −3′ (reverse). The primers for U6 were 5′‐ CCTGCTTCGGCAGCACA −3′ (reverse). The primers for let‐7g were 5′‐GCTGTACAGGCCACTGCCTTGC‐3′ (reverse). These primers were designed and synthesized by Sangon Biotech (China). The qRT‐PCR was performed using the SuperReal PreMix Plus (SYBR Green) (TIANGEN, FP205) and miRNA SuperReal PreMix Plus (SYBR Green) (TIANGEN, FP411) on a 7500 Real‐Time PCR System (ABI, USA). The delta cycle threshold method was used to analyse the relative expression of the target gene.

### 2.7 Verification of the expression of let‐7g and EGF by animal experiments

The mice were randomly divided into two groups (n = 6 per group): control group (C) and burn group (B). The third‐degree burn model was established by the above method. let‐7g and EGF were assayed by qRT‐PCR and western blotting.

### 2.8 Statistical analyses

SPSS25.0 statistical software (SPSS Inc) was used to process all the data, and the experimental results are presented as mean ± standard deviation. The *t* test was used for analysing differences between two groups, and one‐way analysis of variance (ANOVA) was used for differences between multiple groups. *P* < .05 indicated a significant difference.

## 3 RESULTS

### 3.1 The expression of autophagy‐related proteins after intestinal autophagy intervention in severely burned mice

**Figure 1:**
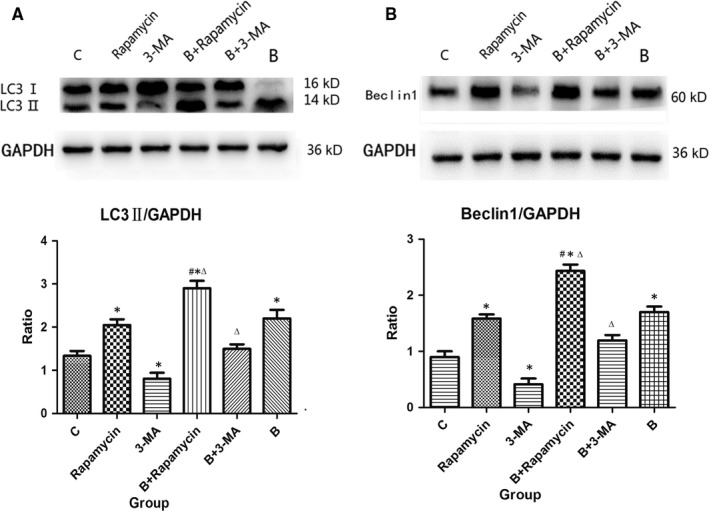
Western blot analysis of intestinal tissue LC3‐II and Beclin1 expression at 24 h post‐burn in mice. (A and B) The expression levels of LC3‐II and Beclin1 were up‐regulated in the Rapamycin, B + Rapamycin and B groups, and down‐regulated in the 3‐MA group when compared with the C group (*P < .05). The levels were more obvious in the B + Rapamycin group and less in the B + 3‐MA group than in the B group (^△^ *P* < .05). The changes in the B + Rapamycin group were more pronounced than in the Rapamycin group (^#^ *P* < .05)

First, the effects of intestinal autophagy intervention on autophagy‐related protein expression were investigated. Western blotting results showed that the expression of the autophagy‐associated proteins LC3‐II and Beclin1 was increased in the Rapamycin group and decreased in the 3‐MA group compared with that in the C group in the intestinal tract. The expression levels of LC3‐II and Beclin1 in the B group were greater than those in the C group. In addition, changes in the B + Rapamycin group were more pronounced than those in the B and Rapamycin groups. On the contrary, the expression levels of LC3‐II and Beclin1 in the B + 3‐MA group were significantly less than those in the B group (all *P* < .05; Figure 1).

### 3.2 Expression of autophagy‐related proteins in IEC cells after autophagy intervention

**Figure 2:**
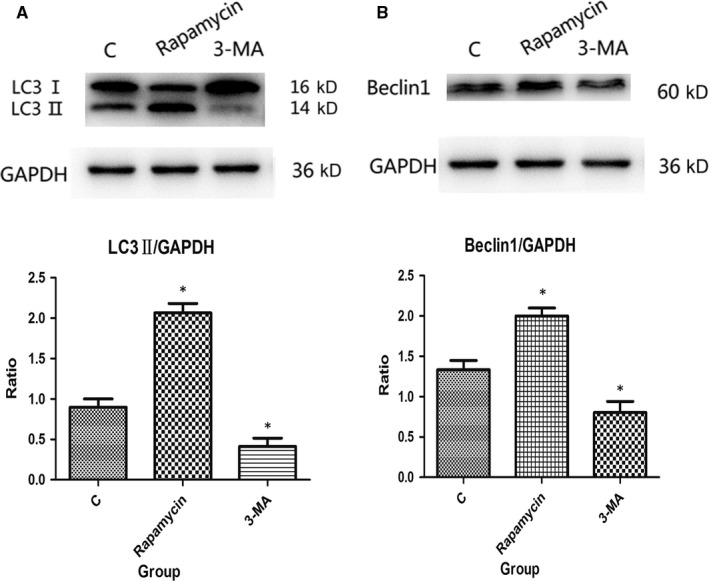
Western blot analysis of LC3‐II and Beclin1 expression in IEC‐6 cells. (A and B) The levels of LC3‐II and Beclin1 were up‐regulated in the Rapamycin group and down‐regulated in the 3‐MA group when compared with the C group (*P < .05)

Notably, the expression levels of autophagy‐related proteins after intestinal autophagy intervention in severely burned mice were markedly increased. To further investigate the protein expression levels in IEC cells after autophagy intervention, Western blotting was performed. The results showed that expression levels of LC3‐II and Beclin1 were greater in the Rapamycin group and less in the 3‐MA group compared with that in the C group in IEC‐6 cells after 24 hours (all *P* < .05; Figure 2).

### 3.3 Change in lncRNA H19 transcription level after intestinal autophagy intervention in severely burned mice

**Figure 3:**
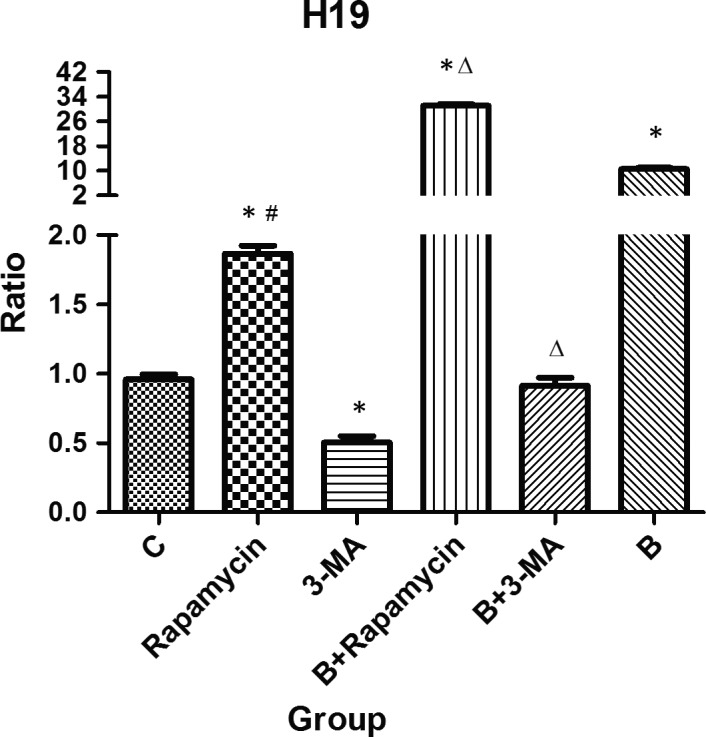
qRT‐PCR analysis of lncRNA H19 expression after the intervention in intestinal autophagy in severely burned mice. The transcription levels of lncRNA H19 were up‐regulated in the Rapamycin, B + Rapamycin and B groups, and down‐regulated in the 3‐MA group when compared with the C group (*P < .05). The levels of those were more obvious in B + Rapamycin group and less in the B + 3‐MA group than in the B group (^△^ *P* < .05). The changes in the B + Rapamycin group were more pronounced than in the Rapamycin group (^#^ *P* < .05)

It has been confirmed that the level of autophagy was increased after a severe burn. However, whether the increase in autophagy level can lead to an increase in the lncRNA H19 transcription level remained unknown. The qRT‐PCR results showed that the transcription levels of lncRNA H19 in Rapamycin, B + Rapamycin and B groups were higher than that in the C group and lower than that in the 3‐MA group. The transcription level of lncRNA H19 in the B + Rapamycin group was higher than that in the Rapamycin group. Compared with the B group, the expression level of lncRNA H19 was up‐regulated and down‐regulated in the B + Rapamycin and B + 3‐MA groups, respectively, and the differences were statistically significant (all *P* < .05; Figure 3).

### 3.4 Changes in lncRNA H19 transcription level in IEC‐6 cells after autophagy intervention

**Figure 4:**
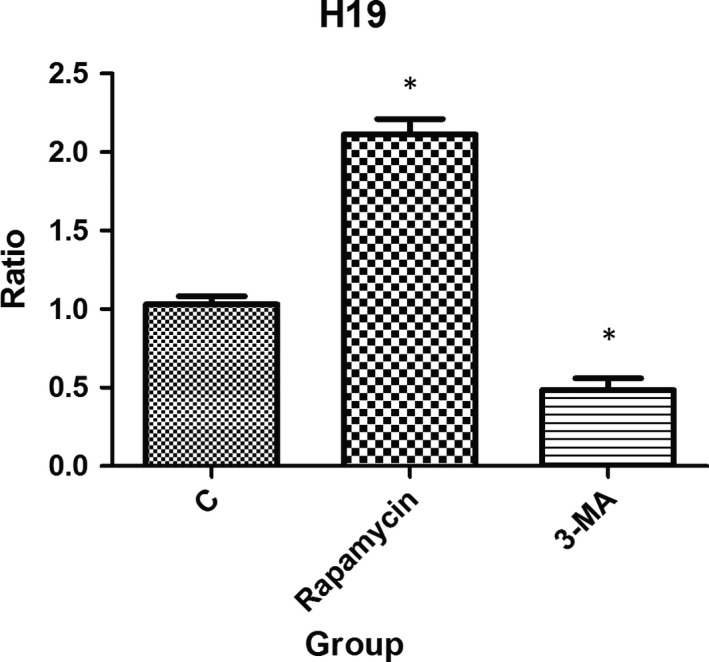
qRT‐PCR analysis of lncRNA H19 transcription level in IEC‐6 cells after autophagy intervention. The transcription levels of lncRNA H19 were up‐regulated in the Rapamycin group and down‐regulated in the 3‐MA group when compared with the C group (*P < .05)

Similarly, the level of lncRNA H19 transcription after intestinal autophagy intervention in severely burned mice was markedly increased. To further investigate the lncRNA H19 transcription level in IEC‐6 cells after autophagy intervention, qRT‐PCR was performed. The results revealed that after the rapamycin treatment, the transcription level of lncRNA H19 in the Rapamycin group was higher than that in the C group. Furthermore, treatment with the autophagy inhibitor 3‐MA resulted in a decrease in the transcription level of lncRNA H19 in the 3‐MA group compared with that of the C group. These differences were statistically significant (all *P* < .05; Figure 4).

### 3.5 Expression levels of EGF in IEC‐6 cells after overexpression of lncRNA H19

**Figure 5:**
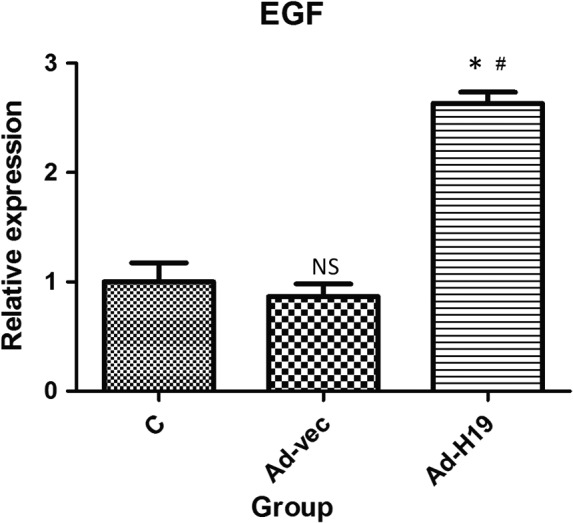
qRT‐PCR analysis of the effect of overexpression of lncRNA H19 on EGF in IEC‐6 cells. Compared with group C and Ad‐vec, the expression level of EGF in the Ad‐H19 group was significantly increased (*P < .05; ^#^ *P* < .05), whereas the difference between the C and Ad‐vec groups was not statistically significant (*P* > .05)

**Figure 6:**
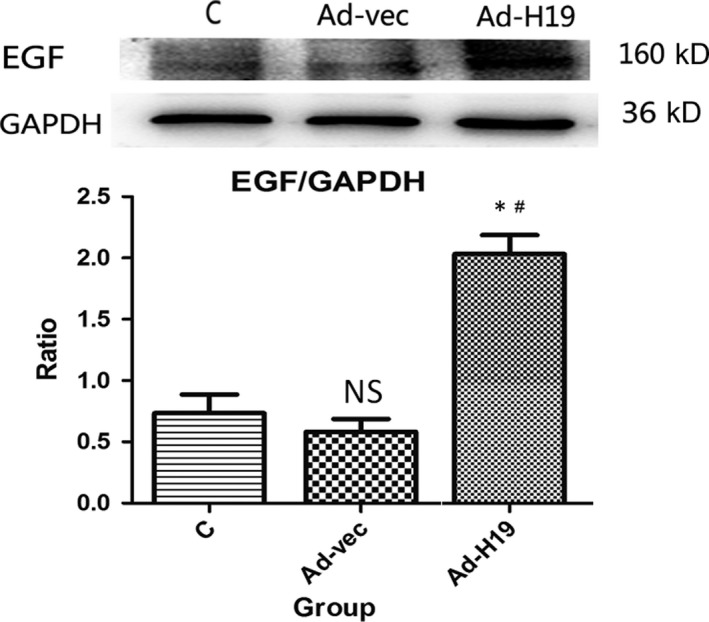
Western blot analysis of the effect of overexpression of lncRNA H19 on EGF in IEC‐6 cells. Compared with the C and Ad‐vec groups, the expression level of EGF in the Ad‐H19 group was significantly increased (*P < .05; ^#^ *P* < .05), whereas the difference between group C and Ad‐vec group was not statistically significant (*P* > .05)

EGF has been shown to be crucial in accelerating the healing of intestinal injury, so we further investigated the regulatory association between lncRNA H19 and EGF. Compared with the C and Adenovirus vector (Ad‐vec) groups, the expression level of EGF in the Ad‐H19 group was significantly increased (all *P* < .05; Figures 5 and 6), whereas the difference between the C and Ad‐vec groups was not statistically significant (*P* > .05).

### 3.6 Expression levels of let‐7g in IEC‐6 cells after overexpression of lncRNA H19

**Figure 7:**
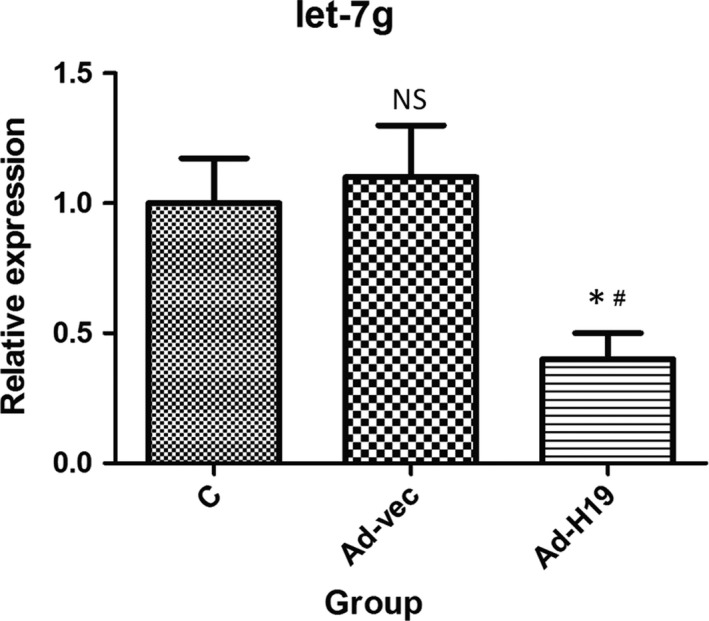
qRT‐PCR analysis of the effect of overexpression of lncRNA H19 on the expression of let‐7g in IEC‐6 cells. Compared with the C and Ad‐vec groups, the expression level of let‐7g in Ad‐H19 group was significantly decreased (*P < .05; ^#^ *P* < .05), whereas the difference between the C and Ad‐vec groups was not statistically significant (*P* > .05)

It has been reported that lncRNAs could sponge miRNAs to regulate mRNA expression by acting as a competing endogenous RNA (ceRNA). We, therefore, assessed for any correlations between lncRNA H19 and let‐7g. We found that the expression level of let‐7g in the Ad‐H19 group was significantly decreased compared with that of the C and Ad‐vec groups (all *P* < .05; Figure 7), whereas the difference between the C and Ad‐vec groups was not statistically significant (*P* > .05).

### 3.7 Effect of let‐7g on EGF protein expression in IEC‐6 cells

**Figure 8:**
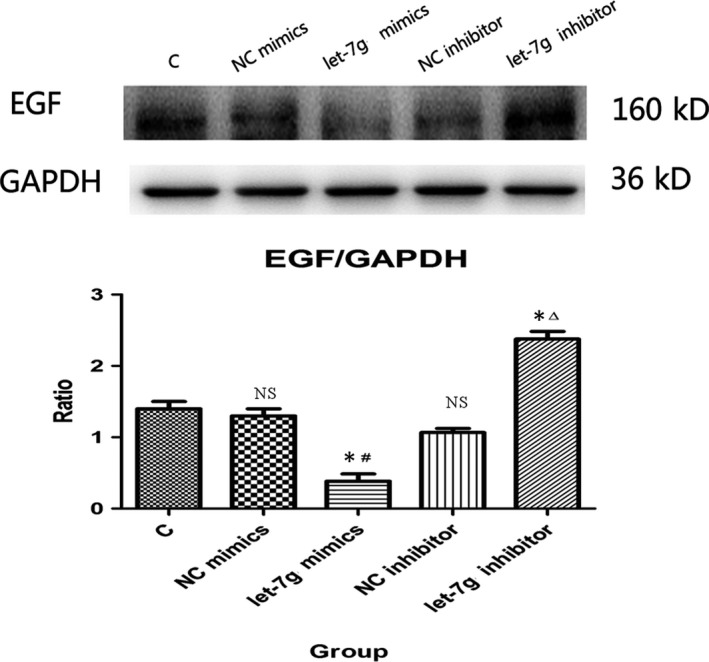
Western blot analysis of the effect of let‐7g on EGF protein in IEC‐6 cells. The expression levels of EGF protein in the let‐7g mimics group were significantly decreased in the IEC‐6 cells when compared with the C group and NC mimics group (*P < .05; ^#^ *P* < .05). Compared with the C and NC inhibitor groups, the expression levels of EGF protein in let‐7g inhibitor group were significantly increased (*P < .05; ^△^ *P* < .05), whereas no statistically significant difference was observed between the C, NC mimics and NC inhibitor groups (*P* > .05)

miRNAs play important roles in disease development by regulating their target genes. To confirm whether let‐7g regulates EGF expression, we transfected let‐7g mimics/inhibitor into IEC‐6 cells. Western blotting results showed that the expression levels of EGF protein in the let‐7g mimics group were decreased in IEC‐6 cells when compared with the control and NC mimics groups. Compared with the control and NC inhibitor groups, the expression levels of EGF protein in the let‐7g inhibitor group were significantly increased (all *P* < .05; Figure 8), whereas no statistically significant difference was observed between the C, NC mimics and NC inhibitor groups (*P* > .05).

### 3.8 Expression levels of let‐7g and EGF in severely burned mice

**Figure 9:**
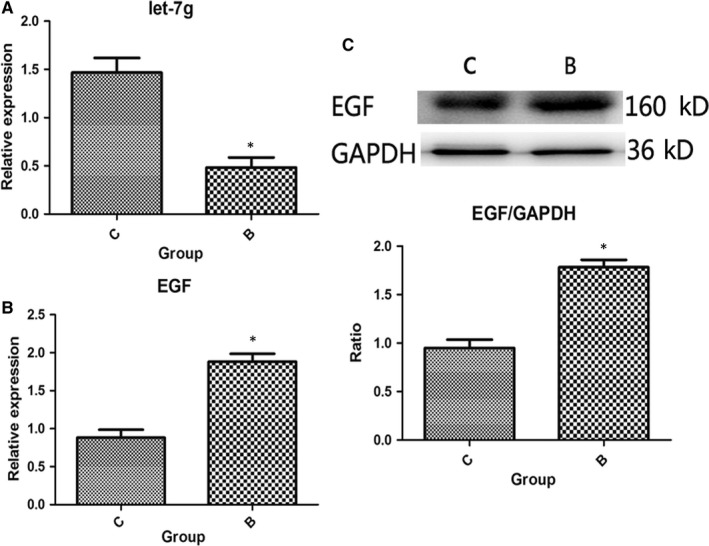
Verification of the expression levels of let‐7g and EGF in in vivo experiments. (A) qRT‐PCR revealed that the expression level of let‐7g in the B group was significantly decreased compared with the control (*P* < .05). (B) qRT‐PCR showed that the EGF expression in the B group was significantly increased compared with the control (*P* < .05). (C and D) Western blotting demonstrated that the expression levels of EGF protein in the B group were significantly increased compared with the control (*P* < .05)

After validating the let‐7g and EGF expression levels in IEC‐6 cells, we evaluated the changes in the expression levels of let‐7g and EGF in severely burned mice. The qRT‐PCR revealed that the expression level of let‐7g and EGF in the B group was significantly decreased and increased, respectively, compared with the control (*P* < .05; Figure 9A‐B). Similarly, Western blotting results showed that the expression levels of EGF protein in the B group were significantly increased compared with the control (*P* < .05; Figure 9C).

## 4 DISCUSSION

To date, the mechanism of early intestinal mucosal damage after severe burns has still not been fully understood. The destruction of the intestinal mucosal protective barrier after a burn is the main cause of intestinal infection. Therefore, the development of methods to reduce intestinal damage after a burn and the identification of effective therapeutic targets to accelerate intestinal repair are important measures for the treatment of intestinal infection. This article focuses on the effect of autophagy and lncRNA H19 after burns in the mouse intestinal tract.

Autophagy is a general term for lysosome‐ or vesicle‐mediated degradation of intracellular components.^35^ Autophagy can provide the compounds needed for cell metabolism and maintain the renewal of some organelles. Based on the different forms of target molecules delivered to lysosomes, autophagy can be divided into macroautophagy, microautophagy, chaperone‐mediated autophagy and atypical autophagy.^36^, ^37^ Autophagy comprises of several stages. The formation of autophagy precursor prolongs the substrates enveloped by autophagy. Next, autophagy bubbles are formed, and autophagy vacuoles and lysosomes fuse to complete substrate degradation. Beclin1 is involved in the formation and initiation of phagocytic vesicles. LC3‐II in the autophagy double‐membrane vesicle appears at the full extension stage, in a gradual process from LC I to LC II.^17^, ^18^, ^20^ The relative expression levels of Beclin1 and LC3‐II reflects cell autophagy activity. Autophagy is involved in the normal growth and development of cells and also plays an important role in coping with various types of stress and diseases.^38^ Autophagy of HT‐29 colon cancer cells is reportedly enhanced after hypoxia,^39^ and autophagy‐related protein expression is increased after intestinal mechanical barrier injury in acute plateau mice. Presently, we used a mouse burn model and IEC‐6 cell culture to study the dynamic changes in autophagy following a burn and in the presence of the autophagy activator rapamycin and the autophagy inhibitor 3‐MA. In mouse intestinal tissue and IEC‐6 cells, autophagy protein levels were elevated after the addition of rapamycin, whereas the opposite was true after the addition of 3‐MA. The level of autophagy in the intestinal tissue of mice increased after a burn and increased even more significantly with the addition of rapamycin, whereas the level of autophagy decreased more after a burn with the addition of 3‐MA.

lncRNAs are a group of endogenous molecules with an approximate length of 200 to 100,000 nucleotides that are transcribed by RNA polymerase. lncRNAs lack specific complete open reading frames and lack a protein‐coding function.^22^ Furthermore, they cover or spread between coding and non‐coding regions. Compared with other non‐coding RNAs, there are many lncRNAs that feature multiple types and multiple modes of action. This results in the regulation of gene expression patterns via multiple pathways and multiple levels. The main regulatory methods include control of the epigenetic inheritance of chromosomes, X chromosome silencing, initiating the regulation of special genes, genomic imprinting, transcriptional activation and the structural framework of nuclear substructure.^40^ These complex regulatory approaches regulate gene expression at the epigenetic, transcriptional and post‐transcriptional levels.^41^

In cardiomyopathy rat models, lncRNA H19 silenced DIRAS3 expression, promoted MTOR phosphorylation and inhibited autophagy in cardiomyocytes.^42^ However, the relationship between lncRNA H19 and autophagy in intestinal tissues after burns remains unclear. In this study, the expression level of lncRNA H19 following a burn in the presence of an activator or inhibitor of autophagy was studied in vivo using a mouse burn model and in vitro in cultured IEC‐6 cells. In both models, the transcription level of lncRNA H19 was increased after the addition of rapamycin but decreased after the addition of 3‐MA. The transcript level of lncRNA H19 was increased in the intestinal tissues of mice after burns. The transcript level of lncRNA H19 was more obvious after burn + Rapamycin administration, whereas the opposite was observed after burn + 3‐MA administration. The results indicated that the increase in intestinal autophagy level could lead to increased transcription of lncRNA H19.

lncRNA plays an extremely important role in growth and development, cell differentiation, proliferation, migration, apoptosis and other processes of the body, as well as in the processes of re‐epithelialization, angiogenesis, scar formation and other regulatory wound healing processes.^43^ H19 is one of the earliest identified imprinted genes. It is located in 11p15.5 of the human chromosome and rat chromosome 7.^44^ Its expression is regulated by the imprinted control region 4 kb upstream,^44^ which is evolutionarily conserved in mammals. lncRNA H19 was discovered earlier and its function is relatively clear. lncRNA H19 is highly expressed in the embryo, but only in the myocardium and skeletal muscle after birth.^32^, ^45^ lncRNA H19 has been closely associated with tumours.^46^ However, its role in intestinal barrier damage and repair after a severe burn remains unknown. The intestinal mucosal repair factor EGF may be regulated by H19, based on evidence in existing literature, including bioinformatics and experimental analyses. Presently, qRT‐PCR and Western blot analyses demonstrated that the increased transcription of H19 can improve the expression level of EGF.

However, the mechanism of its regulatory effect still needs to be confirmed. The available evidence indicates that let‐7g may be involved in the lncRNA H19 regulation of EGF expression. H19 can adsorb the miRNA let‐7 family of molecules.^28^ There are 13 members of the let‐7 family known in humans, with only a few bases differing between them. Mature let‐7 can completely bind to the 3' non‐coding region of the target mRNA to degrade the target mRNA, or it can partially bind to the 3' non‐coding region of the target mRNA to inhibit the translation process of its target mRNA, thus achieving the regulation effect on genes.^31^ Therefore, we first used adenovirus to overexpress lncRNA H19. The use of real‐time fluorescence quantitative PCR demonstrated that the increase in the transcription level of lncRNA H19 can reduce the expression level of let‐7g. Next, let‐7g mimics and a let‐7g inhibitor were overexpressed by transfection. Western blot analyses showed that the expression levels of EGF protein were decreased in the let‐7g mimics group and increased in the let‐7g inhibitor group, indicating that let‐7g inhibited the EGF translation process. Thus, lncRNA H19 may inhibit miRNA let‐7g to increase the expression level of the target protein EGF, but its specific mechanism remains to be determined.

The completion of this study will help to better understand the expression and regulation of autophagy and lncRNA in the process of intestinal mucosal injury repair after burns, and also will elucidate the regulation of autophagy‐mediated lncRNA H19 on intestinal mucosa‐associated repair factor, thus deepening the understanding of the repair process after intestinal mucosal injury. It will also provide a new biomarker for the early diagnosis of intestinal mucosal barrier injury and allow for autophagy and lncRNA to act as new targets for the treatment of intestinal mucosal injury after burns.

## 5 CONCLUSION

**Figure 10:**
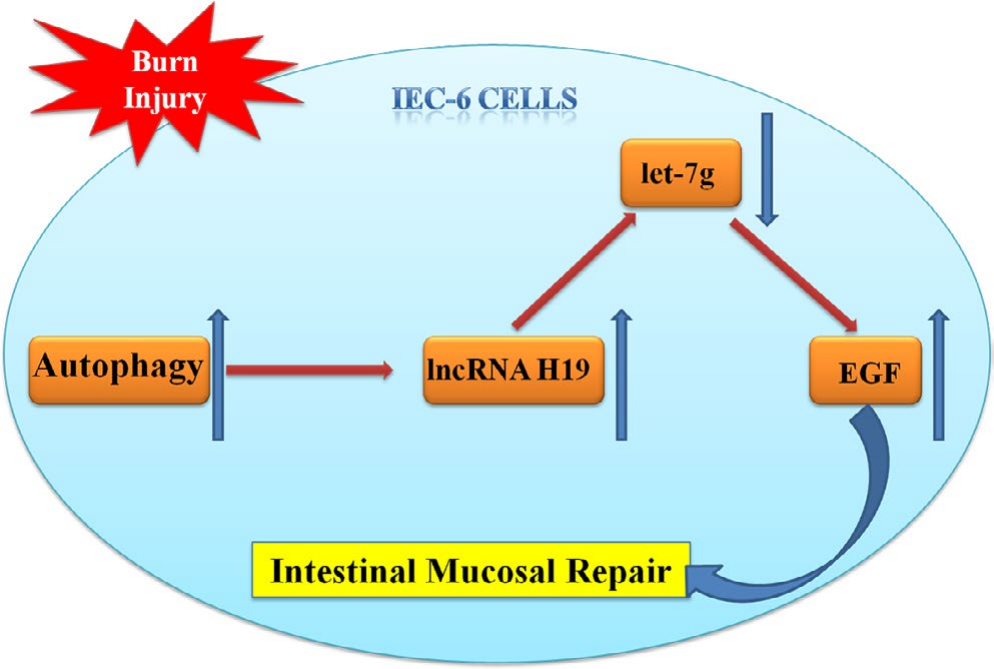
The technical route of this study

The level of autophagy in the intestinal tissue of mice was increased after severe burns. The increase in the autophagy level in the intestinal tract can lead to an increase in lncRNA H19 transcription level. lncRNA H19 enhances the expression of the target gene EGF. lncRNA H19 may enhance the expression of EGF by inhibiting let‐7g, thereby promoting the repair of the intestinal mucosa (Figure 10).

## CONFLICT OF INTEREST

The authors declare no conflicts of financial interests.

## AUTHOR CONTRIBUTION

CJL, MMZ, BZ, YL, WWZ, HY, PSJ and YS designed the experiments. CJL, YL and MMZ conducted the experiments. CJL, BZ,WWZ and HY interpreted and analysed the data. CJL, PZ, DL, YS, HJC and QWC contributed to literature search/analysis tools. CJL and YS made critical revisions. CJL wrote the article.

## Data Availability

The data sets used and/or analysed during the current study are available from the corresponding author on reasonable request.
